# Thriving in Nursing Work: The Association Between Self‐Reports and Biomarkers of Stress, Inflammation and Neuroplasticity

**DOI:** 10.1111/jan.70540

**Published:** 2026-02-11

**Authors:** Judith E. Arnetz, Eamonn Arble, Jackeline Iseler, Michelle Pena, Nicolina Evola, John Vanschagen, John S. Beck, Scott E. Counts, Bengt B. Arnetz

**Affiliations:** ^1^ Department of Family Medicine, College of Human Medicine Michigan State University Grand Rapids Michigan USA; ^2^ Department of Psychology Eastern Michigan University Ypsilanti Michigan USA; ^3^ Trinity Health Grand Rapids Michigan USA; ^4^ Kirkhof College of Nursing Grand Valley State University Grand Rapids Michigan USA; ^5^ Department of Translational Neuroscience College of Human Medicine Grand Rapids Michigan USA; ^6^ Department of Family Medicine and Public Health Sciences, School of Medicine Wayne State University Detroit Michigan USA

**Keywords:** biomarkers, cluster analysis, cortisol, nursing, questionnaire, thriving

## Abstract

**Aim:**

To examine whether self‐reported thriving at work is associated with biomarkers of stress, inflammation, neuroplasticity and neurodegeneration in nurses.

**Design:**

A cross‐sectional study.

**Methods:**

An online questionnaire measuring thriving at work was administered to nurses in a teaching hospital in Michigan, U.S. over 5 weeks in 2024. A subsample of 100 questionnaire respondents provided blood samples for biomarker analysis. Multiple regression was used to identify self‐reported and biomarker predictors of nurse thriving. Cluster analysis was used to distinguish between nurses with high and low levels of thriving based on a combination of self‐report and biomarker data.

**Results:**

Higher self‐reports of individual and work‐related resources predicted higher thriving. Cortisol, a stress hormone, was significantly and inversely associated with thriving. No blood‐based biomarkers of inflammation or neuroplasticity predicted thriving. Neurofilament light chain, a marker of neurodegeneration, was not a direct predictor but modified the effects of interpersonal and work resources on thriving.

**Conclusion:**

Biological markers do play a role in nurses' thriving at work and may contribute important complementary information to that provided by nurse self‐reports.

**Implications for the Profession and/or Patient Care:**

Nurses thrive in a work situation characterised by positive reports of individual, interpersonal and work resources and lower levels of stress. Efforts to enhance thriving could positively impact nurses' well‐being and conditions for providing high‐quality patient care.

**Impact:**

This study addressed the question of whether self‐reported thriving at work among nurses is reflected in biomarkers of stress, inflammation, and neurocognitive health. A profile of high self‐reported work‐related resources and low cortisol distinguished higher levels of nurses' thriving from lower levels. Organisational efforts to enhance nurses' thriving can positively impact nurses' health, their work environment, and patient care.

**Reporting Method:**

We followed the STROBE checklist in reporting this study.

**Patient or Public Contribution:**

No Patient or public contribution.

## Abstract (highlights)

**What Is Already Known?:**

- A growing body of research is focused on factors that contribute to nurses' thriving at work.
- Previous studies have examined biomarkers of stress, inflammation and neurocognitive function as an indication of worker response to their working conditions.
- No studies to date have examined whether nurses' self‐reported thriving could be reflected in biological measures.

**What this paper adds:**

- Higher self‐reports of individual and work‐related resources predicted higher thriving.
- Cortisol, a stress hormone, was significantly and inversely associated with thriving and neurofilament light chain, a marker of neurodegeneration, and modified the effects of interpersonal and work resources on thriving
- Nurses' thriving at work may impact biological markers that may contribute important complementary information to that provided by nurses' self‐reports.

**Implications for practice/policy:**

- Nurses thrive in work environments characterised by positive interpersonal and work resources and lower levels of stress, suggesting that thriving encompasses both psychological/work‐related and physical health.
- Organisational strategies aimed at enhancing nurses' thriving can improve nurse well‐being and conditions for patient care.
- A greater focus on nurses' thriving is needed to help meet the challenges of today's healthcare organisations.

## 1 Introduction

In response to troublesome reports on widespread nurse burnout (Shah et al. 2021; Rink et al. 2023) and an impending shortage in the nursing workforce (Peters 2023; Michaeli et al. 2024), a growing body of research has focused on factors that contribute to nurses' thriving at work (e.g., Jackson 2022; Shen et al. 2024; Zhai et al. 2023; Zhao et al. 2018). This salutogenic approach focuses on factors that support and enhance health and well‐being, as opposed to factors that contribute to disease and ill‐health (Antonovsky 1996; Becker et al. 2010). Thriving at work has been defined as a positive psychological state characterised by two domains, vitality, and learning (Spreitzer et al. 2005). When workers thrive, they feel energised by their work tasks and by continuously learning on the job (Kleine et al. 2019). In nursing, thriving has been positively associated with ‘strength use’, that is, applying one's unique strengths and talents to one's work (Bai and Bai 2024), psychological resilience (Jackson et al. 2007; Shen et al. 2024), and work performance (Shen et al. 2024). Conversely, factors that diminish nurses' thriving include a heavy workload, the emotional burden of nursing work, ethical issues (Jackson et al. 2007), and workplace violence (Zhao et al. 2018; Zhu et al. 2021). Thriving in nursing work has also been associated with authentic nurse leadership (Mortier et al. 2016; Zhu et al. 2021), and research has focused on nurse leader (Frangieh et al. 2023) and healthcare organisation strategies (Moloney et al. 2020) for enhancing nurses' thriving. Thus, the literature has identified individual nurse factors, such as workplace mindfulness (Zhu et al. 2021) and resilience (Shen et al. 2024), as well as organisational factors, such as organisational justice (Zhu et al. 2021) and work engagement (Zhai et al. 2023), that contribute to nurses' thriving at work.

## 2 Background

### 2.1 Biomarkers and Their Connection to Thriving at Work

Since nursing work is widely recognised as inherently stressful and work stress can be measured physiologically (O'Donovan et al. 2013), it is interesting to consider whether thriving in a challenging work environment can also be measured physiologically. McEwen (1998) described the body's adaptation to stress, or allostasis, as the ‘activation of neural, neuroendocrine and neuroendocrine‐immune mechanisms’ (McEwen 1998, 33). If this physiological response is prolonged, such as in the case of chronic stress, the body's cardiovascular, immune, and endocrine systems are dysregulated, resulting in allostatic load (McEwen 2015; Chandola et al. 2019). To better understand the mechanisms by which the body's systems interact with each other and communicate with the brain, McEwen posited that we must study biomarkers (McEwen 2015). Epel et al. (1998) described thriving in response to stress, defining thriving as both psychological and physical health. Physical thriving can be operationalised at the macro level, for example, improved function following injury, as well as at the micro level, for example, hormonal imbalance. They posited that psychological thriving cannot be understood without examination of the physiological correlates of an individual's experiences.

Previous studies have examined biomarkers as an indication of worker response to their working conditions. Levels of dehydroepiandrosterone‐sulfate (DHEA‐S), an anabolic androgen with a protective role, were significantly lower in non‐stressed compared to stressed Swedish workers (Lennartsson et al. 2013). In a large study of flexible work arrangements among over 6000 UK employees, Chandola et al. (2019) reported lower levels of allostatic load index among women with young children who had flexible work arrangements compared to women who did not. A longitudinal study of 122 diverse workers during the COVID‐19 pandemic found that work engagement was positively related to DHEA‐S, measured in hair (Girardi et al. 2024). Among healthcare workers, Mitoma et al. (2008) found that serum levels of brain‐derived neurotrophic factor (BDNF), a marker of neuroplasticity, were higher in hospital employees with higher levels of psychological job stress. Stress in medical residents working in an emergency department was associated with tumour necrosis factor (TNF)‐alpha, a biomarker of inflammation (Arnetz et al. 2017). Resident physicians also showed a reduction in secretion of hair cortisol, a stress hormone, after participating in a mindfulness programme that resulted in improvements in perceived stress, mental health, work‐related well‐being, and thriving at work (Fendel et al. 2020). In a study of 44 female nursing staff, cortisol in saliva was inversely associated with decision authority at work (Harris et al. 2007). Work stress among nurses has been inversely associated with DHEA‐S and positively correlated with interleukin‐6 (IL‐6), a pro‐inflammatory marker (Arnetz et al. 2019).

## 3 Study

In a recent paper, Arnetz et al. (2026) developed and psychometrically evaluated the Thriving in Nursing Questionnaire (THINQ) aiming to measure thriving in nursing work specifically. They found that nurses' thriving was defined by three domains: individual resources, work resources, and interpersonal work resources. The authors hypothesized—but did not test—whether self‐reported thriving, or lack of thriving, would be reflected in biological measures, including biomarkers of stress, inflammation, and neuroplasticity (Arnetz et al. 2026). Building on this previous research, the aims of the current study were to study the associations between the three constructs—individual resources, work resources, and interpersonal work resources—and (1) self‐reported thriving at work; and (2) nurses' biomarkers of stress, inflammation, and neuroplasticity. This study was guided by the following hypotheses:

*Individual resources, work resources and interpersonal work resources will each be positively associated with self‐reported thriving at work*.

*Biomarkers of stress and inflammation will be inversely associated with self‐reported thriving at work*.

*Biomarkers of neuroplasticity will be positively associated with self‐reported thriving at work*.

*A profile of perceived resources and biomarkers of stress and inflammation will identify a group of nurses who report high levels of thriving as compared to those who report low levels*.

## 4 Methods

### 4.1 Study Setting

The study was carried out in the spring of 2024 in a community hospital in Michigan, U.S., which employs approximately 5000 workers.

### 4.2 Study Participants

All registered direct care nurses (*n* = 1100) were invited to participate in the ‘Nurse Thriving and Well‐Being’ study via an email from hospital nurse leadership. The email explained that the purpose of the study was to examine how nurses' personal and work factors affect their well‐being and performance. Nurses were informed that they were being asked to complete an online, anonymous questionnaire to measure their well‐being and performance. Nurses who agreed to participate accessed the electronic Qualtrics questionnaire by using the weblink or by scanning the QR code provided in the email. The first page of the questionnaire stated that participation was voluntary, that individual responses could not be linked to nurses' names or emails, and that nurses were giving their consent to participate by filling out the questionnaire.

### 4.3 Data Collection

#### 4.3.1 Questionnaires

The online questionnaire was active for a 5‐week period from late March to early May 2024. Weekly reminders were posted by nursing leadership on the hospital's nursing Facebook page, and posters advertising the questionnaire were also placed around the hospital to enhance recruitment.

#### 4.3.2 Blood Draw

The final survey question asked for a subsample of 100 questionnaire respondents who were willing to give a blood sample on a single occasion. Nurses willing to participate were asked to provide their contact information on the following page and were informed that researchers would contact them to set up an appointment. The surveys of nurses who volunteered for blood draws were assigned identification numbers in order to link survey responses with blood samples, which were labelled with the corresponding ID numbers. Volunteers were assured that their identifying information would be deleted and replaced with a unique numerical identifier once matched with the nurse's associated data. Questionnaire responses would remain anonymous, and there would be no way to link names and contact information to nurse responses. As thanks for their participation, participants received a $25 gift card. Participants were selected on a first‐come first‐served basis, and blood sampling took place between April 29 and May 3, 2024. Nurses who participated in the blood draw signed a consent form beforehand. Blood samples were collected on site in a hospital lab and were transported to the university for analysis.

### 4.4 Ethical Considerations

The study was approved by the Institutional Review Boards at Michigan State University (Study ID 00009623) and Trinity Health Grand Rapids Hospital (Study ID 24‐0122‐4).

### 4.5 Questionnaire

Questionnaire development and preliminary validation have been described previously (Arnetz et al. 2026). The 30‐item questionnaire was comprised of 21 items concerning individual well‐being (3 items) and perceptions of the nursing work environment (18 items). All 21 items were single‐item measures using visual analog scales (VAS). VAS items were measured on a scale from 0 to 10, with anchor terms very poor—very good (3 items), completely disagree—completely agree (15 items), or never—always (3 items). Following the VAS items were 7 demographic items measuring work unit, work shift, years working as a nurse, age, gender identity, ethnicity, and race. These were followed by a single‐item measure of burnout (Rohland et al. 2004) that asked nurses to rate their level of burnout using a response scale from 1 (‘I enjoy my work. I have no symptoms of burnout’) to 5 (‘I feel completely burned out and wonder if I can go on. I am at the point where I may need some changes or may need to seek some sort of help’). The final question asked for 100 nurse volunteers willing to provide blood samples for measurement of biomarkers.

#### 4.5.1 Independent Variables

Based on exploratory and confirmatory factor analysis (Arnetz et al. 2026), 15 questionnaire items comprised three distinct subscales. *Individual Resources* (3 items) asked nurses to rate their current energy, ability to concentrate, and overall health on VAS scales from 0 (very poor) to 10 (very good). Cronbach's alpha for the subscale was 0.86. The *Work Resources* (6 items) subscale included statements on professional development, necessary skills, necessary unit staffing, unit efficiency, ability to handle tough situations at work, and teamwork. These six items were measured on VAS scales from 0 (completely disagree) to 10 (completely agree), and Cronbach's alpha was 0.83. The *Work Interpersonal Resources* (6 items) subscale included items on a positive unit work climate, supervisor feedback, supervisor inspiration, playing a role in unit decision‐making, satisfaction with salary, and unit psychological safety, that is, ability to bring up problems and tough issues. These six statements were also rated on a VAS scale from 0 (completely disagree) to 10 (completely agree). Cronbach's alpha for the subscale was 0.87. Each subscale was created by summing the scores for its component items, with higher scores representing better perceptions of individual, work, and work interpersonal resources.

#### 4.5.2 Dependent Variable

##### 4.5.2.1 Thriving at Work

In development of the questionnaire (Arnetz et al. 2026), the definition of thriving at work was based on the National Academy of Medicine's (NAM) National Plan for Health Workforce Wellbeing (NAM 2022). Thriving was measured using three items, including feeling fulfilled at work, feeling that work is meaningful, and feeling joyful at work. Those three survey items were therefore summed and analysed for use as a single outcome measure and were not included in the questionnaire's psychometric analysis. Cronbach's alpha for the Thriving subscale was 0.87.

### 4.6 Blood Biomarkers

Serum and plasma specimens were taken from each subject for biomarker analysis. At sample collection, 20 mL blood was drawn by a phlebotomist in two tubes, and the time of day of sample collection was recorded. Each tube was labelled with the ID number assigned to the participant and was appropriately processed on‐site to generate serum and plasma samples, respectively, for each participant. All samples were frozen and stored in a −80°C freezer for later analysis. Following completion of sample collection, samples were transported in travel‐compatible freezers to the university research lab for analysis. Analysis focused on biomarkers of stress (cortisol), anti‐stress or recovery (dehydroepiandrosterone sulfate, DHEA‐S), inflammation (C‐reactive protein CRP, interleukin‐6, IL‐8, IL‐10), neuroplasticity (brain‐derived neurotrophic factor, BDNF, and nerve growth factor, NGF) and neurodegenerative disease (neurofilament light chain, NfL).

All analytes except NfL were measured in serum or plasma samples using human specific 96‐well ELISAs. Single Molecular Array (SIMOA) analysis was used to measure plasma NfL concentrations. The following kits were used to generate data from serum samples: Cortisol (ALPCO #11‐CRLHU‐E01, Salem, NH; Sensitivity = 0.4 μg/dL), CRP (ALPCO #30‐9710S; 0.124 ng/mL), DHEA‐S (ALPCO #11‐DHEHU‐E01; 5 ng/mL), IL‐6 (BioLegend #430507, San Diego, CA; 1.6 pg/mL), IL‐8 (Boster Bio #EK0413, Pleasanton, CA; 1 pg/mL) and IL‐10 (BioLegend #430607; 2 pg/mL). The following kits were used to generate data from plasma samples: BDNF (Boster Bio #EK0307; Sensitivity = 15 pg/mL), NGF (AVIVA #OKEH00186, San Diego, CA; 1.38 pg/mL), NfL (Quanterix #104073, Billerica, MA; 1.38 pg/mL). Duplicate samples were quantified by optical densitometry at 450 nm using a standard curve based on calibrators of known concentration, except in the case of NfL where samples were run on a SIMOA HD‐1 instrument (intra‐assay % CVs ≤ 5.4% for all assays).

### 4.7 Data Analysis

Prior to hypothesis testing, all data were screened for assumptions of multivariate normality, univariate outliers by *z*‐scores < |3.3|, and multivariate outliers (by Mahalanobis distance judged against a critical chi‐square threshold for alpha = 0.001). Descriptive statistics were used to examine nurse‐rated scores for Thriving at work (3 items), the Individual Resources (3 items), Work Resources (6 items), and Work Interpersonal Work Resources (6 items) subscales, as well as for the biomarkers. Bivariate Pearson correlations were used to examine associations between all self‐report and biomarker values.

Hypothesis 1 of the associations among self‐reported resource scales and thriving, controlling for age and years' experience, was tested in *N* = 208 that had complete data, of which two cases were identified as multivariate outliers. Hypothesis 2a and 2b tests of associations with blood‐based biomarkers were assessed in the *n* = 100 subset of cases with relevant measures, of which five cases were identified as outliers in at least one blood assay of interest. All analyses were completed with pairwise deletion, preserving the largest available sample size for each analysis to prioritise external validity and statistical power of each hypothesis test. Analyses were repeated after removing relevant outlier cases to ensure the pattern of effects was similar, albeit potentially underpowered.

#### 4.7.1 Perceived Resources Predicting Thriving

Hypothesis 1 tested perceived Individual Resources, Work Resources, and Interpersonal Resources predicting Thriving, including years of experience and categorical age as covariates in a multiple regression procedure using *F*‐tests for omnibus effects (*α* = 0.05). Summary scores were computed as the mean of responses to each item for each scale. Hypothesis 2a and 2b assessed the association of blood‐based biomarkers with Thriving and potential interaction with perceived resources. Multiple regression models included Individual Resources, Work Resources, Work Interpersonal Resources, blood‐based biomarkers, and interaction terms as predictors of Thriving. Interaction terms that were not significant were removed, and final models were interpreted. Significant interaction effects were decomposed with descriptive correlation analysis stratified at the median level of each blood‐based biomarker. Each blood‐based biomarker was tested separately, and false discovery rate adjustment by the two‐stage sharpened method (Benjamini et al. 2006) was applied for the multiple comparisons made (*q* threshold < 0.05).

#### 4.7.2 Blood‐Based Biomarkers Predicting Thriving

Hypothesis 3 aimed to identify a multivariate profile of combined perceived resources and biomarkers of stress, inflammation and neuroplasticity that could differentiate nurses reporting high thriving as compared to those reporting low thriving. Using all available data (*N* = 208), responses to the Thriving scale were submitted to a k‐means cluster analysis to identify natural sub‐groups within the sample. The predicted group assignment was saved for each case, and subsequently used in a discriminant function analysis to predict ‘high thriving’ and ‘low thriving’ groups by individual resources, work resources, interpersonal work resources, the biomarkers of stress and inflammation, and age and years' experience as covariates. Prior probability of group membership was estimated from the observed frequency of the groups. The model significance was interpreted alongside predicted group accuracy with cross‐validation, and the rank order of predictor loadings in the structure matrix (by convention, >|0.3| indicate meaningful contributions to the discriminant function). Because the discriminant function analysis included blood‐based biomarkers, the analysis was completed with listwise deletion and included *n* = 98 with complete data. To prioritise parsimony for an analysis with this sample size, the model was estimated with standard entry and again with stepwise entry to inform a minimum number of predictors necessary to differentiate the groups.

## 5 Results

A total of 252 surveys were returned out of the 1100 that were distributed, for a 23% response rate (Arnetz et al. 2026). In addition, a subset of 100 of the nurses who responded to the questionnaire also provided blood samples. In the current study, complete self‐report data were available for 208 nurses, and complete biomarker data were available for 100 nurses. A total of 98 nurses had complete self‐report and complete biomarker data. Substantial data were missing for IL‐6 and IL‐8 in the subsample, presumably due to poor sample quality or the current sensitivity limits of the best assays for these markers. Based on concerns of power and validity, these were therefore omitted and are not reported. Table 1 summarises demographic characteristics of the total study sample and for the subset of nurses who provided blood samples for biomarker analysis. In both groups, more than 90% of the nurses were female and White, the majority worked the day shift, and at least half had worked for 10 years or longer as a nurse. The distribution across age categories was similar in both groups, with 25–34 years the largest category (32.7% and 29%, respectively).

**TABLE 1:** Demographics of the total study sample (*N* = 208) and subset that provided blood samples (*n* = 100).

Demographic variable	Total samples		Subset with blood samples	
	Frequency (*n*)	Percent (%)	Frequency (*n*)	Percent (%)
Age				
18–24 years	21	9.8	7	7.0
25–34 years	70	32.7	29	29.0
35–44 years	53	24.8	25	25.0
45–54 years	39	18.2	23	23.0
55–64 years	21	9.8	11	11.0
65–74 years	6	2.8	4	4.0
Prefer not to answer	4	1.9	1	1.0
Total	214	100	100	100.00
Missing	38	15.1	0	0
Gender				
Male	15	7.0	5	5
Female	197	92.1	95	95
Prefer not to answer	2	0.9	0	0
Total	214	100	100	100
Missing	38	15.2	0	0
Race/Ethnicity				
White/Caucasian^a^	201	93.9	95	95
Asian	3	1.4	2	2
Black/African American^a^	1	0.5	1	1
American Indian/Alaska Native	1	0.5	1	1
Prefer not to answer	8	3.7	1	1
Total	214	100	100	100
Missing	38	15.1	0	0
Usual shift				
Day	162	76.1	83	83
Mid‐day	11	5.2	1	1
Night	40	18.8	16	16
Total	214		100	100
Missing	39	15.5	0	0
Years as a nurse				
< 5 years	44	20.8	17	17
5–10 years	62	29.2	23	23
> 10 years	106	50.0	59	59
Total	212	100	99	99
Missing	40	15.9	1	1

*Note:* Demographic description of the original sample (*N* = 208) is summarised from self‐report responses to categorical scales.

^a^ Respondents within this group self‐identified as Hispanic, Latino or of Spanish origin: *n* = 3 Hispanic White Caucasian, and *n* = 1 Hispanic Black/African American.

See Table 2 for a summary of univariate distributions and correlations for all measures of interest, excluding age and years of experience reported as nominal scales. Out of a maximum score of 10, nurses' mean score for Thriving at Work was 7.32 (SD 1.74). Scores on the resource scales were highest on Work Resources (mean 7.88, SD 1.39), with mean scores of 6.99 for both Individual Resources (SD 1.57) and Interpersonal Resources (SD 1.39). Pearson correlations between Thriving and the resource subscales ranged from 0.643 to 0.736 (*p* < 0.05 for all). A significant correlation was also found between Work Resources and Interpersonal Resources (*r =* 0.673). There were no statistically significant correlations between Thriving at Work and any of the biomarkers. Significant correlations were found between Individual Resources and NfL (*r* = 0.230). Among biomarkers, significant correlations were found between cortisol and DHEA‐S (*r* = 0.293), cortisol and NfL (*r* = 0.208), CRP and IL‐10 (*r* = 0.276), CRP and BDNF (*r* = 0.322), and IL‐10 and NfL (*r* = 0.199). Of note, DHEA‐S was excluded from all subsequent biomarker analyses due to its recognised co‐secretion with cortisol (Kamin and Kertes 2017), and the risk that the two biomarkers would steal variance from one another. Cortisol, a stress‐sensitive hormone, has been shown to have a dominant effect over DHEA‐S in studies including both biomarkers (Hirokawa et al. 2018).

**TABLE 2:** Descriptive statistics of study variables.

Variable		*N*	Mean (SD)	Median	Bivariate correlations								
					1	2	3	4	5	6	7	8	9
1	Thriving	208	7.32 (1.74)	7.67									
2	Individual Resources	208	6.99 (1.57)	7.03	0.643*								
3	Work Resources	208	7.88 (1.39)	8.13	0.681*	0.568*							
4	Interpersonal Resources	208	6.99 (1.98)	7.48	0.736*	0.532*	0.673*						
5	Cortisol (μg/dL)	100	6.38 (3.88)	5.08	−0.180	−0.040	−0.179	−0.123					
6	DHEA‐S (μg/mL)	100	1.55 (0.79)	1.51	−0.112	−0.102	−0.079	−0.109	0.293*				
7	CRP (ng/mL)	100	6159.38 (5917.09)	4521.33	−0.013	−0.090	0.028	−0.001	0.175	−0.052			
8	IL‐10 (pg/mL)	100	3.92 (5.31)	2.80	0.150	0.122	0.115	0.097	−0.032	−0.088	0.278*		
9	BDNF (pg/mL)	100	148.67 (92.79)	129.25	0.019	−0.138	0.148	0.075	−0.032	0.086	0.321*	0.049	
10	NfL (pg/mL)	100	5.37 (2.72)	4.78	0.163	0.230*	0.175	0.195	−0.209*	−0.340*	−0.116	0.196	0.079

*Note:* Descriptive statistics and bivariate Pearson correlations are reported for the sample, *N* = 208 responded to the survey and a subset of *n* = 100 provided blood samples for biomarker estimates. Covariates of age and years' experience were reported on ordinal scales, and not included in the summary table.

Abbreviations: BDNF, brain derived neurotrophic factor; CRP, C‐reactive protein; DHEA‐S, dehydroepiandrosterone sulfate; IL‐10, interleukin 10; *M*, mean; NfL, neurofilament light; SD, standard deviation.

* *p* < 0.05.

### 5.1 Perceived Resources Predicting Thriving

Hypothesis 1 tested the association among perceived resources and Thriving (Table 3). Accounting for age (*F*_5,198_ = 1.66, *p* = 0.15, *η*_p_^2^ = 0.04) and years of experience (*F*_1,198_ = 0.40, *p* = 0.53, *η*_p_^2^ = 0.002), unique effects of perceived resources were statistically significant. Greater perceived Individual Resources (*b* = 0.31; *F*_1,198_ = 28.87, *p* < 0.001, *η*_p_^2^ = 0.13), Work Resources (*b* = 0.28; *F*_1,198_ = 13.87, *p* < 0.001, *η*_p_^2^ = 0.07), and Work Interpersonal Resources (*b* = 0.40; *F*_1,198_ = 62.17, *p* < 0.001, *η*_p_^2^ = 0.24) were associated with greater reported Thriving. Among these effects, Work Interpersonal Resources contributed the largest unique effect, followed by Individual Resources and Work Resources. The cumulative model accounted for 67.3% variance in Thriving (adjusted *R* ^2^ = 0.658; *F*_9,198_ = 45.18, *p* < 0.001). Repeating the analysis excluding the cases presenting as multivariate outliers replicated the pattern and magnitude of all effects.

**TABLE 3:** Regression‐based predictors of Thriving.

Predictor	*F*‐test	*p*	*η*_p_^2^	*b*‐weight
Intercept	0.02	0.89	0.00	−0.67
Individual Resources	28.87	< 0.001	0.13	0.31
Work Resources	13.87	< 0.001	0.07	0.28
Interpersonal Resources	62.17	< 0.001	0.24	0.40
Experience	0.40	0.53	0.002	0.09
Age	1.66	0.15	0.04	
18–24 years				1.27
25–34 years				0.65
35–44 years				0.57
45–54 years				0.37
55–64 years				0.69

*Note:* Model *R* ^2^ = 0.673 (adjusted *R* ^2^ = 0.658), *F*_9,198_ = 45.18, *p* < 0.001. Age was reported as an ordinal variable, and age 65–74 years was the reference for b‐weight estimates.

As subsequent hypothesis testing was limited to the sample with biomarker data, this analysis was confirmed to be similar when limited to the *n* = 98 with complete data given this restriction. The model accounted for 70.3% variance in Thriving (adjusted *R* ^2^ = 0.672; *F*_9,88_ = 23.13, *p* < 0.001), and Individual Resources (*b* = 0.32; *F*_1,88_ = 14.29, *p* < 0.001, *η*_p_^2^ = 0.14), Work Resources (*b* = 0.38; *F*_1,88_ = 12.27, *p* < 0.001, *η*_p_^2^ = 0.12) and Interpersonal Resources (*b* = 0.28; *F*_1,88_ = 13.57, *p* < 0.001, *η*_p_^2^ = 0.13) all had comparable effects.

### 5.2 Blood‐Based Biomarkers Predicting Thriving

Hypotheses 2a and 2b tested associations of blood‐based biomarkers with Thriving and the potential to moderate the effects of perceived resources therein. As age and experience were not significant covariates in previous analysis, these were omitted when testing effects of blood‐based biomarkers. All analyses are reported for the subset of participants who additionally had blood collection (*N* = 100). All patterns of effects replicated with exclusion of cases that presented as an outlier to the assays, although the interaction terms were not significant (*q*'s > 0.06) and likely underpowered with the smaller sample size.

Hypothesis 2a considered stress (Cortisol) and inflammation (CRP and IL‐10) markers (Table 4). Higher cortisol was associated with lower reported Thriving (*b* = −0.39, *F*_1,94_ = 9.21, *q* = 0.002; *η*_p_^2^ = 0.09), and modified the effect of Work Resources (*b* = 0.05; *F*_1,94_ = 8.13, *q* = 0.003; *η*_p_^2^ = 0.08). At higher levels of cortisol, the association of Work Resources with Thriving was stronger (*r* = 0.76, *p* < 0.001) as compared to lower levels (*r* = 0.72, *p* < 0.001). Main effects of Individual Resources (*q* = 0.0001) and Interpersonal Resources (*q* = 0.0002) remained significant but were not moderated by cortisol (all ns). The model accounted for 71.3% of variance (*F*_5,94_ = 46.78, *p* < 0.001).

**TABLE 4:** Summary of regression model results predicting thriving for each blood‐based biomarker.

Biomarker	Predictor	Predictor coefficients				Model test	
		*b*	*F*‐test	FDR *q*	*η*_p_^2^	*R* ^2^	*F*‐test
Cortisol	Intercept	**3.20**	**9.98**	**0.0015**	**0.10**	0.713	46.78, *p* < 0.001
	Individual Resources	**0.36**	**21.60**	**0.000116**	**0.19**		
	Work Resources	−0.02	0.01	0.352125	0.00		
	Interpersonal Resources	**0.28**	**17.23**	**0.000168**	**0.16**		
	Cortisol	**−0.39**	**9.21**	**0.0021**	**0.09**		
	Cortisol × Work Resources	**0.05**	**5.79**	**0.003281**	**0.08**		
CRP	Intercept	0.51	0.85	0.171341	0.01	0.683	51.27, *p* < 0.001
	Individual Resources	**0.29**	**13.90**	**0.000452**	**0.13**		
	Work Resources	**0.37**	**12.62**	**0.00057**	**0.12**		
	Interpersonal Resources	**0.28**	**16.59**	**0.000168**	**0.15**		
	CRP	0.00	0.01	0.352125	0.00		
IL‐10	Intercept	0.52	0.90	0.171341	0.10	0.685	51.75, *p* < 0.001
	Individual Resources	**0.29**	**13.80**	**0.000452**	**0.13**		
	Work Resources	**0.37**	**12.68**	**0.00057**	**0.12**		
	Interpersonal Resources	**0.28**	**16.63**	**0.000168**	**0.15**		
	IL‐10	0.01	0.61	0.198587	0.01		
BDNF	Intercept	0.54	0.95	0.171341	0.01	0.684	51.31, *p* < 0.001
	Individual Resources	**0.28**	**12.20**	**0.000587**	**0.11**		
	Work Resources	**0.38**	**12.33**	**0.000587**	**0.12**		
	Interpersonal Resources	**0.28**	**16.64**	**0.000168**	**0.15**		
	BDNF	0.00	0.05	0.33	0.00		
NFL	Intercept	−1.12	0.50	0.210875	0.01	0.705	37.05, *p* < 0.001
	Individual Resources	**0.37**	**19.61**	**0.000137**	**0.17**		
	Work Resources	**0.81**	**12.37**	**0.000518**	**0.13**		
	Interpersonal Resources	−0.04	0.07	0.33075	0.00		
	NFL	0.27	0.97	0.171341	0.01		
	NFL × Work Resources	**−0.10**	**5.37**	**0.014**	**0.06**		
	NFL × Interpersonal Resources	**0.07**	**5.30**	**0.014**	**0.05**		

*Note:* Bivariate interaction terms of each biomarker were tested for each Individual, Work and Interpersonal Resource scale; interaction terms that were not significant were removed from the model. All final models are reported, including adjusted significance testing with False Discovery Rate correction (FDR *q* value). Bolded effects were statistically significant after correction.

There was no evidence for blood‐based biomarkers of inflammation predicting Thriving. CRP was not directly associated with Thriving (*b* = 0.00, *F*_1,95_ = 0.006, *q* = 0.35; *η*_p_^2^ = 0) and did not modify any perceived resource scale effects (all ns). Similarly, IL‐10 was not a significant predictor (*b* = 0.01, *F*_1,95_ = 0.61, *q* = 0.20, *η*_p_^2^ = 0.01) and did not moderate effects (all ns). Each model accounted for approximately 68% of the variance in Thriving (*F*_4,95_ = 51.27 and 51.75, respectively; both *p* < 0.001) that was largely due to the main effects of Individual Resources (*q's* < 0.0005), Work Resources (*q's* = 0.0006) and Interpersonal Resources (*q*'s = 0.0002).

Hypothesis 2b considered blood‐based biomarkers of neural plasticity and neurodegeneration, for which there was mixed evidence (Table 4). NfL modified the association of Work Resources (*b* = −0.10, *F*_1,88_ = 5.37, *q* = 0.01; *η*_p_^2^ = 0.06) and Work Interpersonal Resources (*b* = 0.07, *F*_1,88_ = 5.30,*q* = 0.01; *η*_p_^2^ = 0.05) with Thriving. At higher risk for neurodegeneration (i.e., higher NfL levels), the associations with Work (*r* = 0.72, *p* < 0.001) and Interpersonal Resources (*r* = 0.70, *p* < 0.001) were relatively lessened as compared to lower levels of NfL (*r* = 0.75 and 0.75, respectively; both *p* < 0.001). The main effect of NfL was not significantly associated with Thriving (*b* = 0.27, *F*_1,88_ = 0.97, *q* = 0.17; *η*_p_^2^ = 0.01) independent of perceived resources. The cumulative model accounted for 71% of variance (*F*_6,88_ = 37.05, *p* < 0.001). BDNF was unrelated to Thriving (*b* = 0.00, *F*_1,95_ = 0.05, *q* = 0.33; *η*_p_^2^ = 0.001) and did not modify any effect of perceived resources (all ns). The model accounted for 68.4% of variance (*F*_4,95_ = 51.31, *p* < 0.001), largely due to the main effects of perceived resources (all *q* < 0.0006).

### 5.3 A Profile to Differentiate Thriving

We aimed to combine the various predictors of interest to describe a profile that could differentiate high thriving from low thriving. First, the Thriving scale was identified to have subgroups of respondents with a k‐means cluster analysis, which identified 2 groups in the responses. Group 1 identified 152 individuals (Thriving = 6.67–10.0; Median = 8.08, *M* = 8.17) and group 2 identified 56 individuals (Thriving = 0.60–6.57; Median = 5.40, *M* = 5.01). Based on the pattern of responses, group 1 was labelled as High Thriving and had significantly higher reported thriving than group 2 (*F*_1,206_ = 383.52, *p* < 0.001).

The predicted group assignments were saved for subsequent discriminant function analysis for cases that also had blood‐based biomarker data (*n* = 98: *n* = 77 High Thriving and *n* = 21 Low Thriving). Following standard entry with all predictors, the discriminant function was statistically significant (Wilks' λ = 0.42; *χ* ^2^_10_ = 79.95, *p* < 0.001). The model had excellent cross‐validated accuracy of 91.8%, and accuracy was good for both High Thriving individuals (97.4%) and Low Thriving individuals (71.4%). The structure matrix indicated the multivariate combination of high Interpersonal Resources (0.71), Work Resources (0.66), Individual Resources (0.51), and low cortisol (−0.30) meaningfully contributed to the differentiation of High Thriving from Low Thriving. All other structure matrix loadings were below |0.3|, see Table 5. Repeating the analysis with stepwise entry to identify a parsimonious model resulted in five entry steps, and all steps were significant (Wilks' *λ* = 0.44–0.58; all *p*'s < 0.001). The final model step retained 5 predictors (Wilks' *λ* = 0.44; *χ* ^2^_5_ = 77.75, *p* < 0.001) and had 91.8% cross‐validated accuracy (98.7% High Thriving; 66.7% Low Thriving). The retained predictors in the final model identified High Thriving with the following order of entry: high Interpersonal Resources, low cortisol, high Work Resources, low NfL and low CRP. Although NfL and CRP were contributing unique variance to the discriminant function to be selected for the stepwise procedure, the final resulting structure matrix loadings were below the conventional threshold |0.3| suggesting a modest contribution to the discriminant function (Table 5). Therefore, results of both models support a profile to identify High Thriving individuals primarily by high Interpersonal and Work Resources, and low Cortisol.

**TABLE 5:** Results of the stepwise discriminant function analysis predicting High Thriving as compared to Low Thriving.

Predictor	Standardised canonical coefficient	Structure matrix loading
Interpersonal Resources	0.681	**0.740**
Work Resources	0.478	**0.687**
Cortisol	−0.454	**−0.312**
CRP	−0.278	−0.132
Nfl	−0.343	0.032
Individual Resources		0.350
BDNF		0.098
IL‐10		−0.075
Age		0.056
Years' Experience		0.100

*Note:* Standardised canonical coefficients are estimated only for predictors retained in the final stepwise model that emphasised parsimony. Structure matrix loadings are reported for all items in the stepwise procedure. By convention, structure matrix loadings > |0.3| are considered meaningfully contributing to the discriminant function (emphasised with bolding for predictors also selected in the stepwise procedure).

Abbreviations: BDNF, brain derived neurotrophic factor; CRP, C‐reactive protein; IL‐10, interleukin 10; NfL, neurofilament light.

## 6 Discussion

### 6.1 Resources as Predictors of Thriving at Work

This study aimed to identify self‐reported factors and biomarkers associated with thriving at work. The first hypothesis, that self‐reported individual and work‐related resources would predict self‐reported thriving at work, was fully supported. Greater perceived resources were associated with greater reported thriving, with each type of resource contributing to the variance in thriving. These findings support previous research suggesting that both individual nurse factors (Zhu et al. 2021; Shen et al. 2024) as well as organisational factors (Zhu et al. 2021; Zhai et al. 2023), contribute to nurses' thriving at work. With regard to individual resources, workplace mindfulness has been identified as a factor in nurses' thriving, suggesting that nurses who are mindful are more self‐aware and have a better ability to concentrate and manage stress (Zhu et al. 2021). This is in line with the self‐reported ability to concentrate in this study's Individual Resources subscale. Zhu et al. (2021) contend that nurses who are mindful differ from nurses working on ‘auto pilot’ (p. 445), who focus primarily on completing tasks to reduce one's workload rather than concentrating closely on each individual task. Thriving at work has also been associated with feeling energised by one's work tasks (Kleine et al. 2019), supporting the energy item in the Individual Resources subscale. Resilience, the ability to adapt to difficult or adverse situations (Shen et al. 2024), has also been associated with nurses' thriving (Jackson et al. 2007; Shen et al. 2024). In the current study, resilience was measured as the ability to handle a tough situation at work, which is an item in the Work Resources subscale. However, it has been suggested that nurses with high levels of resilience are generally more positive, with better emotional insight and life balance (Jackson et al. 2007), qualities that are closely aligned with the overall health item in the Individual Resources subscale.

While prior studies have identified organisational factors that contribute to thriving (Bai and Bai 2024; Mortier et al. 2016; Zhai et al. 2023; Zhu et al. 2021), few studies have identified nursing‐specific elements of thriving such as those in this study's Work Resources subscale. The subscale encompasses items closely related to nursing practice, including nurse professional competence, patient care skills, adequate unit staffing, unit efficiency, ability to handle tough situations at work, and unit teamwork. Bai and Bai (2024) describe the importance of strength use, that is, the individual application of an individual nurse's ‘unique strengths and talents’ (p. 2) as a strategy that healthcare organisations could employ to maximise nurses' individual skills and enhance their thriving. Other organisational factors that contribute to nurses' thriving include organisational justice (Zhu et al. 2021), work engagement (Zhai et al. 2023), and authentic nurse leadership (Mortier et al. 2016; Zhu et al. 2021). Several of these are captured by the current study's Work Interpersonal Resources measure. A positive work climate, ability to bring up problems and tough issues, and satisfaction with salary are three subscale items closely related to organisational justice. Playing a role in decision‐making is an element of work engagement, while supervisor feedback and inspiration are associated with authentic nurse leadership.

### 6.2 Biomarkers as Predictors of Thriving at Work

There were no statistically significant correlations between Thriving at Work and any of the biomarkers. However, regression analyses revealed that cortisol, a stress marker, was an inverse predictor of Thriving, in partial support of our second hypothesis. Previous research on cortisol in nurses found that it was positively associated with self‐reported stress (Harris et al. 2007; Yang et al. 2001) and inversely associated with decision authority (Harris et al. 2007). Cortisol also modified the effect of Work Resources on Thriving so that the higher the cortisol level, the stronger the association between Work Resources and Thriving. This was an unexpected and potentially important finding. The Work Resources subscale concerns aspects of nursing related to work performance, such as professional competence, necessary nursing skills, unit staffing, and unit efficiency. At higher levels of cortisol, the association between Work Resources and Thriving was stronger than at lower levels, suggesting that Work Resources may serve as an important buffer for nurses with higher levels of this stress hormone.

The role of cortisol is especially interesting given the nature of nursing work, primarily characterised by working shifts (Rosa et al. 2019). The majority of the nurses participating in this study worked primarily day shifts and had 10 years of experience or more. Cortisol is known to be highest in the morning upon awakening and declining over the course of the day (de Vries et al. 2022). This raises questions as to whether lower levels of cortisol reflect higher thriving, or whether higher thriving—perhaps attributed to years of experience and a supportive environment—leads to low cortisol levels.

None of the biomarkers of inflammation predicted Thriving directly, thus there was no support for that hypothesis. A systematic review on the association between biomarkers and well‐being found a slight role for serotonin, cortisol, and inflammatory markers. The studies in the review utilised general measures of well‐being and not a specific measure such as thriving at work (de Vries et al. 2022). Regarding biomarkers of neuroplasticity and neurodegeneration, neither BDNF nor NfL was associated with Thriving, and neither was an independent predictor of Thriving in regression analysis. NfL moderated the association between both Work Resources and Work Interpersonal Resources with Thriving, with higher values of NfL associated with weaker associations with both resource measures. Higher levels of BDNF were reported in hospital employees with higher levels of work stress (Mitoma et al. 2008). However, that study was not focused on thriving at work.

The third hypothesis was that it would be possible to identify two distinct profiles of nurses reporting high versus low thriving based on perceived resources and biomarkers of stress, inflammation, and neuroplasticity. The hypothesis was partially supported in that the profile identified that was able to differentiate between groups was comprised of a combination of Interpersonal Resources, Work Resources, and lower levels of cortisol. NfL and CRP did contribute modestly to the discriminant function, but below conventional thresholds. Biomarkers involved in the inflammatory cascade, for example, CRP, have been associated with changes in memory and learning. Immune proteins are required for many key neural processes and inflammation can result in impairment in such processes (Donzis and Tronson 2014). The findings that both low CRP and low NfL were associated with the thriving profile suggest that thriving is indeed associated with not only self‐reported work factors, for example, interpersonal work resources, but, indeed, decreased inflammation and neuronal strain. It is also notable that only work‐related resources contributed significantly to a high thriving profile, not individual resources. Thus, self‐reports of energy, ability to concentrate, and overall health had less of an influence on thriving than work resources (e.g., necessary unit staffing), work interpersonal resources (e.g., receiving constructive feedback from one's supervisor), and biomarkers.

Few other studies have attempted to identify profiles of nurses who thrive at work. Wu et al. (2025) distinguished three groups of nurses based on low, medium, and high strength use, finding that greater strength use was associated with greater thriving at work. To the best of our knowledge, no previous studies have included biomarkers in profile analysis related to thriving at work.

This study identified both self‐reported factors as well as biological markers that are associated with thriving at work among nurses. Self‐reported Individual, Work, and Interpersonal Work Resources were all significant predictors of thriving. Among biomarkers, cortisol, a biomarker that is reflective of chronic stress processes, was inversely and significantly associated with thriving. However, cortisol moderated the effect of Work Resources on Thriving, and NfL moderated the effect of both Work Resources and Interpersonal Resources on thriving. Moreover, a profile of high work interpersonal resources, work resources, and low cortisol distinguished a higher level of nurse thriving from a lower level. Of interest is the finding that the profile of thriving nurses included low levels of markers of inflammation (CRP) and neurodegeneration (NfL). Immune proteins play a key role in learning and memory (Donzis and Tronson 2014). In our study, the thriving concept implies learning and development, for example, developing professional competence in the Work Resources subscale. Thus, although our biomarker findings are below the normal statistical threshold, they highlight the need to consider the impact of nurse thriving, or lack of thriving, on brain health and development.

### 6.3 Study Limitations

This study utilised a cross‐sectional design, and it is therefore not possible to determine causal relationships between self‐report measures, biomarkers, and nurses' thriving. The study was conducted at a single hospital, and results may not be generalizable to nurses in other geographic areas. The majority of participating nurses were female and White, and results may not be generalizable to nurses of other genders and races. Blood samples on the subsample of 100 nurses were drawn in close proximity to the time of questionnaire completion, but not on the same day. Thus, biomarker results may have been influenced by work environment or external events that occurred in the intervening time. Blood samples in the study were drawn at various times of the day based on each nurse's schedule and availability. It was therefore not possible to standardise the blood draw process to a particular time of day. This may have affected levels of cortisol, a stress hormone known to fluctuate based on the time of day (de Vries et al. 2022). However, the influence of any diurnal fluctuation was minimised since biomarker values were studied on an aggregate level. Moreover, low cortisol was consistently identified in the discriminant analysis models as a meaningful contributor to thriving among nurses. Finally, we did not measure nurses' height and weight, and body mass index has been shown to vary in women across the weight spectrum (Schorr et al. 2015).

## 7 Conclusion

This study found that nurses thrive in work environments characterised by positive interpersonal and work resources and lower levels of stress, suggesting that thriving encompasses both psychological/work‐related and physical health. Findings also indicate that nurses' thriving at work may be reflected in biological markers, which may therefore contribute important complementary information to that provided by nurse self‐reports.

### 7.1 Implications for Nursing Practice, Policy and Research

Results from this study have important implications for nursing practice and its future. While findings underscore the influence of a positive work environment on nurses' thriving, the fact that low levels of thriving are associated with biomarkers of stress, inflammation, and neurocognitive health must not be overlooked. Neurodegeneration, defined as the progressive loss of neuronal structure and function, is to a degree a normal part of the ageing process and is affected by an array of genetic, environmental, inflammatory and other factors (Marzola et al. 2023). If lack of thriving at work can contribute to cognitive decline among nurses, the potential negative implications for the nursing profession and for patient care are tremendous. Conversely, conditions that promote neuroplasticity, such as learning new skills, have been shown to potentially slow down or reverse neurodegeneration and cognitive decline (Marzola et al. 2023).

Healthcare organisations that make efforts to improve interpersonal and work‐related resources, decrease stress, and provide opportunities for skills' development can contribute to nurses' thriving and enhance conditions for patient care. Future research should further examine whether measuring biomarkers of stress, inflammation and neuroplasticity can be useful to nurses and their workplaces as a measure of current conditions as well as potential effects of workplace interventions on nurses' thriving and cognitive health. With a shortage of nurses and an ageing nursing workforce worldwide (WHO 2025), the need to support nurses and help them to thrive at work has never been greater.

## Funding

This work was supported by Contributory Research Funds, Trinity Health, Grand Rapids.

## Conflicts of Interest

The authors declare no conflicts of interest.

## Data Availability

The data that support the findings of this study are available from the corresponding author upon reasonable request.
